# Evaluation of reference genes for quantitative real-time PCR normalization in the scarab beetle *Holotrichia oblita*

**DOI:** 10.1371/journal.pone.0240972

**Published:** 2020-10-21

**Authors:** Minghui Xie, Yongzhi Zhong, Lulu Lin, Guangling Zhang, Weihua Su, Wanli Ni, Mingjing Qu, Haoliang Chen

**Affiliations:** 1 Institute of Plant Protection and Agro-Products Safety, Anhui Academy of Agricultural Sciences, Hefei, Anhui, China; 2 Crop Research Institute, Anhui Academy of Agricultural Sciences, Hefei, Anhui, China; 3 Shandong Peanut Research Institute, Qingdao, Shandong, China; Ghent University, BELGIUM

## Abstract

Quantitative real-time polymerase chain reaction (qPT-PCR) is commonly used to analyze gene expression, however, the accuracy of the normalized results is affected by the expression stability of reference genes. *Holotrichia oblita* (Coleoptera: Scarabaeidae) causes serious damage to crops. Reliable reference genes in *H*. *oblita* are needed for qRT-PCR analysis. Therefore, we evaluated 13 reference genes under biotic and abiotic conditions. RefFinder provided a comprehensive stability ranking, and geNorm suggested the optimal number of reference genes for normalization. *RPL13a* and *RPL18* were the most suitable reference genes for developmental stages, tissues, and temperature treatments; *RPL13a* and *RPS3* were the most suitable for pesticide and photoperiod treatments; *RPS18* and *RPL18* were the most suitable for the two sexes. We validated the normalized results using odorant-binding protein genes as target genes in different tissues. Compared with the selected suitable reference genes, the expression of *OBP1* in antennae, abdomen, and wings, and *OBP2* in antennae and wings were overestimated due to the instability of *ACTb*. These results identified several reliable reference genes in *H*. *oblita* for normalization, and are valuable for future molecular studies.

## Introduction

Quantitative real-time polymerase chain reaction (qRT-PCR), based on fluorescent signal monitoring, is commonly used for quantitative analysis of genes [1–3]. In most molecular studies, such as RNA sequencing (RNA-Seq) or RNA interference (RNAi), qRT-PCR is required to confirm accurate transcript changes of the target genes [4]. The reliability of qRT-PCR results is influenced by the availability of the reference genes [5]. The minimum requirements for qRT-PCR indicate that the effectiveness of reference genes as internal controls must be verified by corresponding experimental design [6]. Housekeeping genes used as reference genes without experimental validation can lead to poor normalization. Shi et al. [7] found that significant differences in the expression of *HSP23* in *Bradysia odoriphaga* could not be detected under different temperatures when using the reference gene *ACTb*. The RNA polymerase Ⅱ gene, which is not a housekeeping gene, had more stable expression than classical housekeeping genes in different human tissues [8]. An idealized reference gene with absolutely stable expression in all conditions could not be found. Expression levels can fluctuate because of various factors [7–10]. The use of inappropriate reference genes can bias quantitative results. Specific genes normalized by a single reference gene are not credible without proper validation [11, 12]. The stability of reference genes under specific experimental conditions must be evaluated to prevent nonbiological variations or errors [13].

*Holotrichia oblita* Faldermann (Coleoptera: Scarabaeidae) is widely distributed in China and causes serious damage to crops, forests, and lawns [14, 15]. *H*. *oblita* adults stay underground during the day, while flying, feeding, and mating occur at night. Larvae feed on plant roots and remain underground during their development. *H*. *oblita* has a broad host range, extended feeding period, and cryptic habits [16]. Chemical pesticides are often used to kill larvae, but these can lead to soil pollution. The use of pheromone-baited traps can reduce larval populations by trapping adults. qRT-PCR has been applied to quantitative studies of olfactory genes to increase understanding of the odor recognition mechanism of *H*. *oblita* [17–21]. The reference genes for *H*. *oblita*, however, have not been evaluated under corresponding experimental conditions. Previously, a single housekeeping gene was randomly used for normalization.

The stability and effectiveness of reference genes for *H*. *oblita* need to be systematically evaluated. We assessed 13 candidate reference genes that involved several factors. These included developmental stage, tissue, sex, temperature, pesticide treatment, and photoperiod. We used the geNorm, NormFinder, BestKeeper, ΔCt method, and RefFinder to identify the stability of candidates. We studied the expression profile of two tissue-specific genes across tissues, which were standardized by the selected reference genes and a commonly used reference gene.

## Materials and methods

### Insects rearing

We collected *H*. *oblita* in Feixi County (117°60′E, 31°39′N), Hefei, Anhui, China, in May 2018. No permits were required for the described study, which complied with all relevant regulations. Adults of both sexes were caught from fields at night and then placed in plastic boxes (60 × 50 × 50 cm) [16]. The bottom of the box was covered with a 20-cm deep soil layer with a moisture content of 15−18%. About 200 adults were housed in each box and the sex ratio was approximately 1:1 [22]. The adults were fed with fresh elm leaves (*Ulmus pumila*). We collected eggs every week and placed them in the box with the same soil. After the eggs hatched, larvae were fed with slices of fresh potato. When the larvae reached the 2nd instar, each larva was put in a separate cup. All of the insects were reared in a walk-in chamber under constant conditions of 25 ± 1°C with 60 ± 5% relative humidity and a 8:16 h (L:D) photoperiod.

### Sample collection and treatment

We evaluated the candidate reference genes under the following settings: developmental stage, tissue, sex, temperature, pesticide treatment, and photoperiod. After being processed under each experimental condition, all samples were immediately put into liquid nitrogen and stored at -70°C. Each treatment had three biological replicates.

#### Developmental stages

The developmental stages included eggs (100 per sample), 1st instar larvae (6 per sample), 2nd instar larvae (1 per sample), 3rd instar larvae (1 per sample), pupae (1 per sample), and adults (1 male and 1 female per sample).

#### Tissues

Male and female adults were dissected into six body parts (antenna, head without antenna, thorax, abdomen, leg, and wing) respectively. One hundred pairs of antenna were separated and pooled as one sample. Samples of the head without antenna, abdomen, and thorax were obtained from two individuals. Leg samples were obtained from 12 individuals and wing samples were obtained from 6 individuals.

#### Temperatures

The 3rd instar larvae of *H*.*oblita* were exposed to 4°C, 10°C, 20°C, or 30°C for 2 h. The surviving individuals at each temperature treatment were collected and frozen. There was one individual larva per sample.

#### Pesticide treatments

We used clothianidin and bifenthrin insecticides in this study. They were dissolved in acetone at 2000 mg/L and 100 mg/L to produce stock solutions. The stock solutions were diluted with deionized water and used to treat soil containing 3rd instar larvae and the tests were scored at 48 h for larval mortality. At 48 h, the LC_50_ concentrations of clothianidin and bifenthrin were 44.668 and 0.875 mg per kg of soil, respectively. The 3rd instar larvae were then placed into soil with the LC_50_ concentrations for 48 h at 25°C. We collected the surviving larvae and used one individual for each sample.

#### Sexes

Three pairs of male and female adults were caught on the night of May 2, which is the local early emergence period. We used one individual for each sample.

#### Photoperiods

We used the laboratory-reared adults for the photoperiod experiments. Newly emerged *H*.*oblita* adults were immediately placed at five photoperiods, including 0:24 h (L:D), 6:18, 12:12, 16:8, and 24:0. One pair of mated adults was put into a transparent plastic box and exposed to one of the five photoperiods randomly. After 7 d, one pair of adults was taken from each photoperiod and constituted one sample.

### Total RNA extraction and cDNA synthesis

We used the MiniBEST Universal RNA Extraction Kit (TaKaRa, Dalian, China) to extract total RNA for all of the noted samples after being ground in liquid nitrogen. The purity and concentration of each RNA sample were checked by NanoVue Plus (GE Company, Fairfield, CT, USA). We used the OD value at a 260/280 nm ratio between 1.85 and 2.10 was used for further cDNA synthesis. Total RNA (1 μg per sample) was reverse transcribed following the manufacturer’s instructions for the PrimeScript^®^RT Reagent Kit with gDNA Eraser (TaKaRa, Dalian, China). We checked the cDNA concentrations on the CFX96 System using RPS6 as a reference and adjusted the Ct value of all samples to approximately 18. Then, the cDNA was stored at -20°C until used.

### Primer design for candidate reference gene

We selected 13 reference genes commonly used for insect research. They included glyceraldehyde-3-phosphate (*GAPDH*), beta-actin (*ACTb*), six ribosomal protein genes (*RPL13a*, *RPL18*, *RPS18*, *RPS6*, *RPS3*, and RPL28), syntaxin-6 (*SYN6*), beta-tubulin (*TUBb*), alpha-tubulin (*TUBa*), ubiquitin-conjugating enzyme (*UBC*), and succinate dehydrogenase (*SDHA*) (Table 1). Because of the stable expression profiles, we evaluated those genes as candidate reference genes in insects [7, 18, 23–26]. The primers of all genes were designed by Primer 5.0 with an optimum Tm of 60 ± 2°C, lengths between 19 to 25 bp, and a PCR product size of 112−198 bp (Table 1). The accession numbers and primers of the genes are listed in Table 1.

**Table 1:** Information about two target genes and 13 candidate reference genes in *Holotrichia oblita*.

**Accession number**	**Gene Name**	**Primers (5’→3) (F: Forward; R: Reverse)’**	**Product Length (bp)**	**Efficieny (E) (%)**	**Regression coefficient (R**^**2**^**)**
GQ856258	Odorant-binding protein 1 (*OBP1*)	F: TTGCGTTGCTCAAACTGGA	194	94.559	0.997
		R: TCTGCTTTATCCTTGTATTCGTCT			
GQ856257	Odorant-binding protein 2 (*OBP2*)	F: ATTTTGTTGTATTTGCTGCATTG	167	99.071	0.991
		R: TGTCGGGTATCTGTTCCTTCAT			
MT213595	Ribosomal protein L13a (*RPL13a*)	F: GAAAGAGGCAAGCAAGCATT	165	100.559	0.997
		R: CCAACCGACTTCGTGAGACA			
MT213596	Ribosomal protein L18 (*RPL18*)	F: CGACCAAAGGATATGGGATG	198	102.752	0.998
		R: GGACCAAAATGTTTCACTGCT			
MT213597	Ribosomal protein S18 (*RPS18*)	F: GCATGAAGAAAATTCGTGCTC	112	102.235	1.000
		R: TTAGATACACCGACTGTGCGAC			
MT213598	Ribosomal protein S6 (*RPS6*)	F: GTATGGGAGCAGAAGTAGAGGC	143	99.893	0.999
		R: CGATAACAGAAGACGGACACG			
MT213599	Ribosomal protein S3 (*RPS3*)	F: ACGACTACGTTGATACGGCTAC	146	102.981	0.999
		R: GGGTTCCACGACGGATACA			
MT213560	Syntaxin-6 (*SYN6*)	F: CGAAATTGATAGTCCTCAAAGG	168	103.025	0.999
		R: TCTAGCATTACTGCTTGCTCATC			
MT213561	Beta-tubulin (*TUBb*)	F: TATGGGCACATTACTCATCTCAA	125	91.331	0.999
		R: AGGGTGGCGTTGTATGGTTC			
MT213562	Alpha-tubulin (*TUBa*)	F: ATACGACCGCCATTGCTGA	163	97.125	0.997
		R: CCATACCTACTTCCTCGTAATCCT			
MT213563	Ubiquitin-conjugating enzyme (*UBC*)	F: CTTTTGTACGAGTAGTTCACCCTAT	160	93.095	1.000
		R: CATTATGACTGCTTCCACCGT			
MT213564	Ribosomal protein L28 (*RPL28*)	F: AAATCGGTTGGCATAATAGATG	158	99.120	0.999
		R: CAGGCGTTTCAGTTTATACAGG			
MT213565	Succinate dehydrogenase (*SDHA*)	F: AAGCCCTAAAAGATCCATTCTC	160	100.682	0.991
		R: GCCATCGGTTCTAAGTCGG			

The melting curve and standard curve were drawn to check the specificity and amplification efficiency. We generated the standard curve by a serial 10-fold dilution of cDNA and calculated the efficiency value (E) of all primers by the formula: E = (10^[−1/slope]^−1)×100 [27, 28].

### qRT-PCR

Each amplification reaction (25 μL) contained 12.5 μL SYBR Premix (Takara Bio, Dalian, China), 2 μL cDNA, 1 μL of each primer (10 μM), and 8.5 μL ddH_2_O. According to the MIQE guidelines, we performed qRT-PCR on CFX96 System (Bio-Rad, Hercules, CA, USA) [6]. The qRT-PCR amplification conditions were set as follow: 95°C for 30 s, followed by 45 cycles at 95°C for 30 s, 58°C for 30 s, and 72°C for 30 s. We performed each treatment with three biological samples and each sample had two technical replicates.

### Stability of candidate reference genes

We used five algorithms to evaluate the stability of the candidate reference genes: RefFinder [29], ΔCt method [30], BestKeeper [28], geNorm [31], and NormFinder [32]. The RefFinder program provided a proper weight for each gene and generated a comprehensive ranking. The geNorm determined the optimal reference gene number by calculating the pairwise variation (V_n_/V_n+1_). The stability measure (M value) of gene expression calculated by geNorm proposed 1.5 as a cut-off line. A value less than 1.5 meant that this reference gene was stably expressed. Generally, lower values calculated by these algorithms indicated higher stability.

### Validation by two target genes

We used two odorant-binding protein genes (*OBP1* and *OBP2*) to verify the stability of the reference genes [33]. We used the optimum single reference gene *RPS13* (RefFinder), the best reference gene pair *RPL13a/RPL18* (geNorm), and a normally used reference gene *ACTb* to calculate the relative expression of *OBP1* and *OBP2* in *H*. *Oblita* [17, 19–21]. We calculated the relative transcript levels of the two target genes according to the 2^–ΔΔCT^ formula and conducted the significance analysis by Tukey’s b test (P = 0.05) across different tissues by SPSS 16.0 (SPSS Inc., 2007, Chicago, IL, USA).

## Results

### Primer amplification efficiency and specificity

Table 1 provides descriptions of the gene name, designed primer pair, product length, and primer amplification efficiency of all genes. We calculated the amplification efficiency by the slope of the standard curves. The efficiency values ranged between 91.33% (*TUBb*) and 103.03% (*SYN6*), with all regression coefficient (R^2^) values > 0.99. We evaluated the primer specificity of all genes by the melting curve. The melting temperatures ranged from 79.00°C (*RPL28*) to 85.00°C (*TUBb*) with a single sharp peak, which confirmed gene-specific amplification.

### Cycle threshold (Ct) values and variations in candidate reference genes

**Fig 1:**
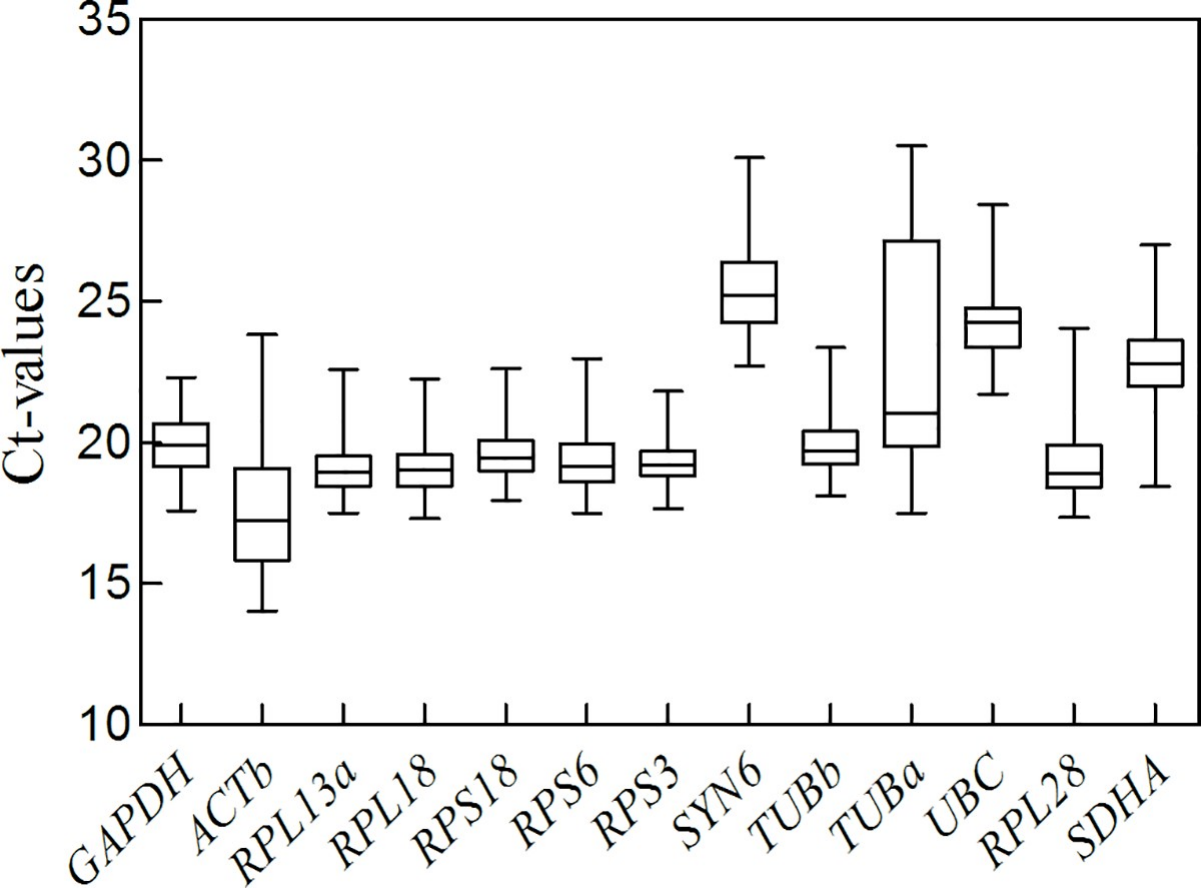
Cycle thresholds (Ct) values of the 13 candidate reference genes for *H*. *oblita*. Each box includes the percentiles that ranged from 25th (lower edge) to75th (upper edge). The whisker caps depict the minimum and maximum data. The median is denoted by a horizontal line inside the box.

For each candidate reference gene, we analyzed Ct values to reveal the level of transcription (Fig 1). Under all of the experimental conditions, raw Ct values of reference genes varied from 14.04 (*ACTb* among different developmental stages) to 30.51 (*TUBa* among different temperatures). *ACTb*, with the lowest median Ct value (17.23), had the highest expression among the genes, whereas *SYN6* with the highest Ct values (25.22) had the lowest expression. *RPS18* had the smallest variance, indicating that it was the most stable, whereas *TUBa* had the highest variance, indicating that it was the most variable.

### Evaluation of candidate reference genes

#### Biotic experimental conditions

**Fig 2:**
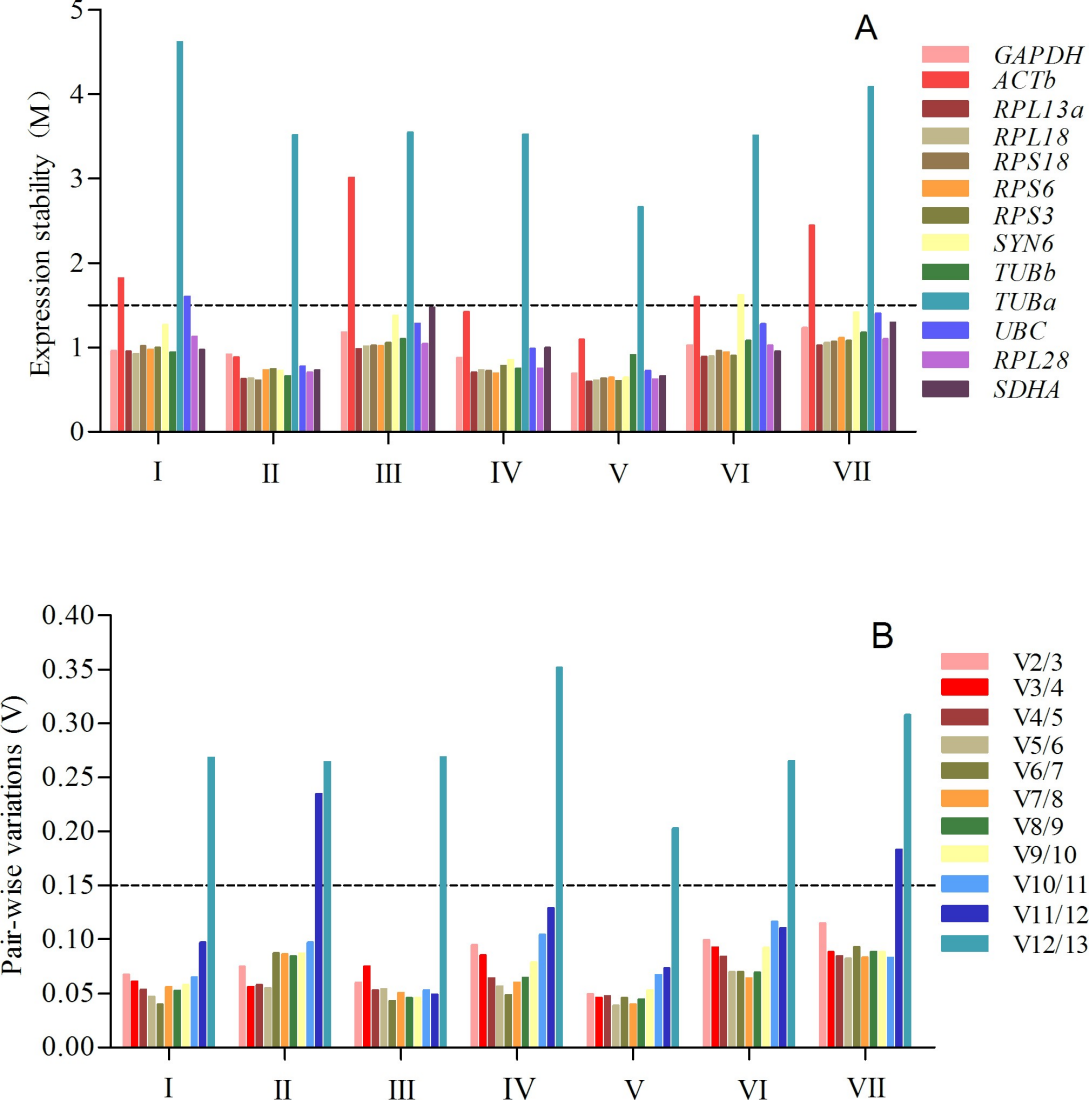
Expression stability (A) and pairwise variation (B) analysis by geNorm for the 13 reference genes in *H*. *oblita*. (Ⅰ) developmental stages, (Ⅱ) tissues, (Ⅲ) sexes, (Ⅳ) temperatures, (Ⅴ) pesticides, (Ⅵ) photoperiods, and (Ⅶ) total samples. (A) Expression stability: The value of V_n_ /V_n+1_ < 0.15 means that the additional reference genes could not significantly optimize the normalization. (B) Pairwise variation: A M < 1.5 is considered to be adequate for normalization.

**Fig 3:**
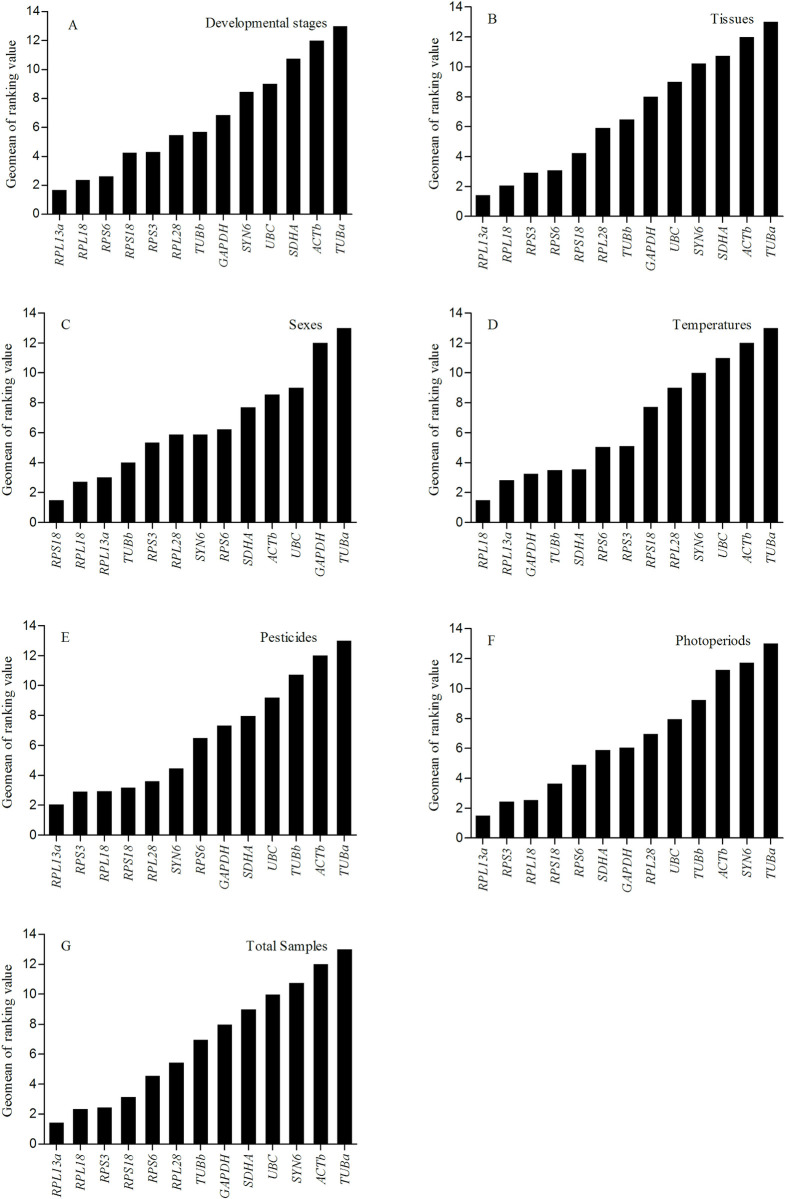
Comprehensive stability of the 13 reference genes in *H*. *oblita* analyzed by RefFinder. The ranking value is listed across the following: (A) developmental stages, (B) tissues, (C) sexes, (D) temperatures, (E) pesticides, (F) photoperiods, and (G) total samples. A lower Geomean value indicates more stability.

As shown in Table 2, geNorm ranked *RPL13a* and *RPL18* as the most stable reference genes for normalization across different development stages. The M values, provided by geNorm, of *ACTb*, *TUBa*, and *UB*C were greater than the critical value of 1.5, and they were considered unsuitable as reference genes (Fig 2A). *RPL13a* and *RPS6* were recommended as the most stable reference genes by NormFinder, while in the BestKeeper ranking, they were *RPS3* and *RPL18* (Table 2). *RPS6* and *RPL13a* had the greatest stability by the ΔCt method (Table 2). The comprehensive ranking provided by RefFinder from the highest to lowest was as follows: *RPL18*, *RPL13a*, *GAPDH*, *TUBb*, *SDHA*, *RPS6*, *RPS3*, *RPS18*, *RPL28*, *SYN6*, *UBC*, *ACTb*, and *TUBa*. The pairwise values of V_2_/V_3_ calculated by geNorm below 0.15 indicated that two reference genes were enough for accurate normalization (Fig 2B). Therefore, *RPL18* and *RPL13a* were demonstrated to be the best reference genes across the developmental stages of *H*. *oblita* (Figs 2B and 3A).

**Table 2:** Stability ranking of candidate reference genesunder biotic conditions, using four statistical algorithms.

Biotic conditions	Rank	GeNorm		NormFinder		BestKeeper		ΔCt	
		Gene	Stability	Gene	Stability	Gene	Stability	Gene	Stability
**Developmental stages**	1	*RPL13a*	0.212	*RPL13a*	0.074	*RPS3*	0.477	*RPS6*	0.696
	2	*RPL18*	0.212	*RPS6*	0.075	*RPL18*	0.502	*RPL13a*	0.701
	3	*RPS6*	0.223	*RPS18*	0.085	*GAPDH*	0.542	*RPS18*	0.723
	4	*RPS18*	0.248	*RPL18*	0.123	*RPL13a*	0.560	*RPL18*	0.731
	5	*RPL28*	0.271	*TUBb*	0.149	*RPL28*	0.577	*TUBb*	0.751
	6	*TUBb*	0.289	*RPL28*	0.151	*UBC*	0.592	*RPL28*	0.752
	7	*RPS3*	0.302	*RPS3*	0.244	*TUBb*	0.593	*RPS3*	0.780
	8	*SYN6*	0.348	*SYN6*	0.301	*RPS6*	0.597	*SYN6*	0.851
	9	*GAPDH*	0.388	*GAPDH*	0.376	*RPS18*	0.617	*GAPDH*	0.878
	10	*UBC*	0.435	*SDHA*	0.427	*SYN6*	0.797	*UBC*	0.965
	11	*SDHA*	0.494	*UBC*	0.463	*SDHA*	0.924	*SDHA*	0.997
	12	*ACTb*	0.613	*ACTb*	0.823	*ACTb*	1.126	*ACTb*	1.421
	13	*TUBa*	1.060	*TUBa*	2.417	*TUBa*	2.739	*TUBa*	3.520
**Tissues**	1	*RPL13a*	0.213	*RPL13a*	0.119	*RPS3*	0.574	*RPL13a*	0.978
	2	*RPL18*	0.213	*RPS3*	0.183	*RPS6*	0.668	*RPL18*	1.013
	3	*RPS18*	0.236	*RPL18*	0.201	*RPL18*	0.669	*RPS6*	1.016
	4	*RPS6*	0.250	*RPS18*	0.216	*RPL13a*	0.747	*RPS18*	1.020
	5	*RPS3*	0.280	*RPS6*	0.218	*RPS18*	0.759	*RPL28*	1.041
	6	*RPL28*	0.309	*TUBb*	0.235	*TUBb*	0.790	*RPS3*	1.053
	7	*TUBb*	0.404	*RPL28*	0.259	*RPL28*	0.877	*TUBb*	1.098
	8	*GAPDH*	0.490	*GAPDH*	0.399	*GAPDH*	1.040	*GAPDH*	1.176
	9	*UBC*	0.566	*UBC*	0.530	*UBC*	1.042	*UBC*	1.284
	10	*SYN6*	0.643	*SYN6*	0.657	*SDHA*	1.099	*SYN6*	1.376
	11	*SDHA*	0.736	*SDHA*	0.659	*SYN6*	1.231	*SDHA*	1.469
	12	*ACTb*	1.090	*ACTb*	1.973	*ACTb*	2.484	*ACTb*	3.010
	13	*TUBa*	1.468	*TUBa*	2.376	*TUBa*	2.871	*TUBa*	3.543
**Sexes**	1	*RPL18*	0.301	*RPS18*	0.053	*RPS3*	0.423	*RPS18*	0.608
	2	*RPS18*	0.301	*RPL13a*	0.091	*TUBb*	0.506	*RPL13a*	0.624
	3	*RPL13a*	0.351	*RPL18*	0.116	*RPS6*	0.506	*RPL18*	0.634
	4	*RPL28*	0.377	*TUBb*	0.146	*ACTb*	0.600	*TUBb*	0.662
	5	*SYN6*	0.416	*SYN6*	0.179	*RPS18*	0.619	*RPL28*	0.703
	6	*UBC*	0.464	*RPL28*	0.200	*RPL18*	0.674	*SYN6*	0.722
	7	*SDHA*	0.536	*RPS6*	0.207	*RPL13a*	0.688	*SDHA*	0.726
	8	*TUBb*	0.590	*SDHA*	0.230	*SYN6*	0.809	*RPS6*	0.730
	9	*RPS6*	0.657	*RPS3*	0.238	*SDHA*	0.813	*RPS3*	0.746
	10	*RPS3*	0.723	*UBC*	0.306	*RPL28*	0.845	*UBC*	0.774
	11	*ACTb*	0.781	*ACTb*	0.426	*UBC*	0.902	*ACTb*	0.881
	12	*GAPDH*	1.027	*GAPDH*	0.461	*GAPDH*	0.937	*GAPDH*	0.916
	13	*TUBa*	1.498	*TUBa*	2.422	*TUBa*	2.307	*TUBa*	3.512

For tissue samples, *RPL13a* and *RPL18* were considered suitable reference genes using geNorm, and the M values for all reference genes were below 1.5 except for *TUBa* (Fig 2A, Table 2). The most stable reference genes were *RPL13a* according to NormFinder and the ΔCt method (Table 2). *RPS3* ranked first in BestKeeper was and ranked second in NormFinder (Table 2). The general ranking, according to RefFinder, was as follows: *RPL13a*, *RPL18*, *RPS3*, *RPS6*, *RPS18*, *RPL28*, *TUBb*, *GAPDH*, *UBC*, *SYN6*, *SDHA*, *ACTb*, and *TUBa* (Fig 3B). On the basis of the pairwise results calculated by geNorm, *RPL13a* and *RPL18* were selected for accurate normalization (Figs 2B and 3B).

For different sexes of adults, the top four of the stable ranking were the same using NormFinder and ΔCt (Table 2). *RPS18*, *RPL13a*, and *RPL18* were recommended as the best reference genes by geNorm, NormFinder, and the ΔCt method; however, in BestKeeper, they were *RPS3*, *TUBb*, and *RPS6* (Table 2). On the basis of the four statistical formulas, *TUBa* was confirmed as the most unstable gene under the three biotic factors (Table 2). GeNorm indicated that *TUBa* and *ACTb* were the most unstable genes because the M values were greater than 1.5 (Fig 2A). As shown in Fig 3C, RefFinder provided the ranking as *RPS18*, *RPL18*, *RPL13a*, *TUBb*, *RPS3*, *RPL28*, *SYN6*, *RPS6*, *SDHA*, *ACTb*, *UBC*, *GAPDH*, and *TUBa* from most stable to most unstable. According to the pairwise value of geNorm, *RPS18* and *RPL18* were the most suitable reference genes across sexes (Figs 2B and 3C).

#### Abiotic experimental conditions

For different temperatures, *RPL18* and *RPL13a* were ranked first and third, respectively, by BestKeeper and ΔCt, and they were the top two by geNorm (Table 3). However, NormFinder showed that *SDHA* was the most stable reference gene (Table 3). The least stable five reference genes were the same as those calculated by the four algorithms with the sequence being *RPL28*, *SYN6*, *UBC*, *ACTb*, and *TUBa* (Table 3). According to the result of geNorm, the pairwise values from V_2_/V_3_ to V_11_/V_12_ were below the 0.15 cutoff line and all gene M values were below 1.5 except for *TUBa* (Fig 2A and 2B). RefFinder ranked *RPL18* and *RPL13a* as the top two, so they were evaluated as the genes most suitable for temperature treatments (Figs 2B and 3D).

**Table 3:** Stability ranking of candidate reference genes under abiotic conditions using four statistical algorithms.

Abiotic conditions	Rank	GeNorm		NormFinder		BestKeeper		ΔCt	
		Gene	Stability	Gene	Stability	Gene	Stability	Gene	Stability
**Temperatures**	1	*RPL13a*	0.243	*SDHA*	0.097	*RPL18*	0.599	*RPL18*	0.922
	2	*RPL18*	0.243	*TUBb*	0.116	*GAPDH*	0.634	*TUBb*	0.941
	3	*RPS6*	0.287	*GAPDH*	0.116	*RPL13a*	0.672	*RPL13a*	0.953
	4	*SDHA*	0.330	*RPS3*	0.119	*RPS3*	0.678	*GAPDH*	0.959
	5	*TUBb*	0.350	*RPL18*	0.185	*TUBb*	0.684	*SDHA*	0.971
	6	*RPS3*	0.368	*RPS6*	0.218	*RPS6*	0.735	*RPS6*	0.978
	7	*GAPDH*	0.381	*RPL13a*	0.241	*RPS18*	0.781	*RPS3*	0.999
	8	*RPS18*	0.418	*RPS18*	0.366	*SDHA*	0.789	*RPS18*	1.016
	9	*RPL28*	0.464	*RPL28*	0.518	*RPL28*	0.885	*RPL28*	1.129
	10	*SYN6*	0.538	*SYN6*	0.573	*SYN6*	0.975	*SYN6*	1.266
	11	*UBC*	0.659	*UBC*	0.881	*UBC*	1.151	*UBC*	1.603
	12	*ACTb*	0.813	*ACTb*	0.991	*ACTb*	1.183	*ACTb*	1.816
	13	*TUBa*	1.397	*TUBa*	3.166	*TUBa*	3.387	*TUBa*	4.613
**Pesticides**	1	*RPL18*	0.146	*RPL13a*	0.067	*RPS18*	0.539	*RPL13a*	0.597
	2	*RPL28*	0.146	*RPS3*	0.078	*SYN6*	0.544	*RPS3*	0.601
	3	*RPL13a*	0.159	*RPS6*	0.105	*RPS3*	0.579	*RPL18*	0.607
	4	*SYN6*	0.181	*RPS18*	0.140	*GAPDH*	0.606	*RPL28*	0.623
	5	*RPS18*	0.213	*RPL18*	0.186	*RPL18*	0.658	*RPS18*	0.632
	6	*RPS3*	0.231	*RPL28*	0.200	*RPL13a*	0.684	*RPS6*	0.641
	7	*SDHA*	0.267	*SYN6*	0.224	*RPL28*	0.698	*SYN6*	0.643
	8	*GAPDH*	0.291	*UBC*	0.241	*SDHA*	0.702	*SDHA*	0.662
	9	*RPS6*	0.324	*SDHA*	0.251	*UBC*	0.756	*GAPDH*	0.695
	10	*UBC*	0.373	*GAPDH*	0.294	*TUBb*	0.785	*UBC*	0.725
	11	*TUBb*	0.445	*TUBb*	0.461	*RPS6*	0.824	*TUBb*	0.910
	12	*ACTb*	0.524	*ACTb*	0.648	*ACTb*	1.147	*ACTb*	1.094
	13	*TUBa*	0.853	*TUBa*	1.826	*TUBa*	1.461	*TUBa*	2.664
**Photoperiods**	1	*RPL13a*	0.330	*RPL13a*	0.145	*RPS18*	0.545	*RPL13a*	0.886
	2	*RPL18*	0.330	*RPS3*	0.165	*RPS3*	0.630	*RPL18*	0.893
	3	*RPS3*	0.337	*RPL18*	0.197	*GAPDH*	0.692	*RPS3*	0.903
	4	*RPS6*	0.374	*RPS6*	0.212	*UBC*	0.692	*RPS6*	0.943
	5	*SDHA*	0.415	*RPS18*	0.242	*RPL13a*	0.763	*SDHA*	0.950
	6	*RPS18*	0.440	*SDHA*	0.264	*RPL28*	0.791	*RPS18*	0.961
	7	*RPL28*	0.475	*GAPDH*	0.344	*RPL18*	0.849	*RPL28*	1.021
	8	*GAPDH*	0.506	*RPL28*	0.355	*SDHA*	0.855	*GAPDH*	1.024
	9	*TUBb*	0.550	*TUBb*	0.433	*RPS6*	0.897	*TUBb*	1.080
	10	*UBC*	0.638	*UBC*	0.583	*TUBb*	1.052	*UBC*	1.274
	11	*ACTb*	0.766	*SYN6*	0.913	*ACTb*	1.383	*ACTb*	1.597
	12	*SYN6*	0.877	*ACTb*	0.915	*SYN6*	1.521	*SYN6*	1.619
	13	*TUBa*	1.282	*TUBa*	2.382	*TUBa*	2.488	*TUBa*	3.502

For pesticide treatments, *RPL13a* was ranked first by NormFinder and ΔCt, whereas in geNorm the best gene was *RPL18* (Table 3). GeNorm results indicated that *RPL18* and *RPL28* were the best reference genes (Table 3). According to geNorm, the results of pairwise values and M values were similar to those of the temperature treatments. *ACTb* and *TUBa* were demonstrated to be the most unstable genes using four analysis tools (Fig 2A). According to RefFinder’s online tool, the integrated ranking was *RPL13a*, *RPS3*, *RPL18*, *RPS18*, *RPL28*, *SYN6*, *RPS6*, *GAPDH*, *SDHA*, *UBC*, *TUBb*, *ACTb*, and *TUBa* (Fig 3E). Combining the results of pairwise values by geNorm, we selected *RPL13a* and *RPS3* to be the most reliable reference genes (Figs 2B and 3E).

For different photoperiods, the top two ranked genes were *RPL13a* and *RPL18* by geNorm and ΔCt, whereas *RPL13a* and *RPS3* were ranked as the top two by NormFinder, *RPS18* and *RPS3* were ranked as the top two by BestKeeper (Table 3). According to the four algorithms, *ACTb*, *SYN6*, and *TUBa* were the most unstable candidate reference genes, and their M values were all higher than 1.5 (Table 3, Fig 2A). In addition, geNorm provided the pairwise value that V_n_/V_n+1_ were all below the 0.15 cutoff, except for V_12_/V_13_, which indicated that two reference genes were sufficient across the photoperiod treatments (Fig 2B). RefFinder indicated the ranking as *RPL13a*, *RPS3*, *RPL18*, *RPS18*, *RPS6*, *SDHA*, *GAPDH*, *RPL28*, *UBC*, *TUBb*, *ACTb*, *SYN6*, *and TUBa* (Fig 3F). *RPL13a* and *RPS3* were considered to be the most suitable reference genes across different photoperiods (Figs 2B and 3F).

#### Total samples

For all of the experimental samples, *RPL13a* and *RPL18* were the best reference genes in the stable ranking based on geNorm and ΔCt methods (Table 4). *RPL13a* and *RPS6* were ranked the top two by NormFinder, whereas they were *RPS3* and *RPS18* by BestKeeper (Table 4). *ACTb* and *TUBa* had M values above 1.5 by geNorm analysis, but were ranked as least stable according to the four methods (Fig 2A). The general ranking generated by RefFinder was *RPL13a*, *RPL18*, *RPS3*, *RPS18*, *RPS6*, *RPL28*, *TUBb*, *GAPDH*, *SDHA*, *UBC*, *SYN6*, *ACTb*, and *TUBa* (Fig 3G). According to the results of the pairwise values, the two genes *RPL13a* and *RPL18* were the best reference genes for all samples (Figs 2B and 3G).

**Table 4:** Stability ranking of candidate reference genes for total samples using four statistical algorithms.

Rank	GeNorm		NormFinder		BestKeeper		ΔCt	
	Gene	Stability	Gene	Stability	Gene	Stability	Gene	Stability
1	*RPL13a*	0.301	*RPL13a*	0.197	*RPS3*	0.611	*RPL13a*	1.018
2	*RPL18*	0.301	*RPS6*	0.208	*RPS18*	0.667	*RPL18*	1.055
3	*RPS3*	0.351	*RPS3*	0.238	*RPL18*	0.718	*RPS18*	1.066
4	*RPS18*	0.377	*RPS18*	0.255	*RPL13a*	0.723	*RPS3*	1.076
5	*RPL28*	0.416	*RPL18*	0.268	*RPL28*	0.807	*RPS6*	1.098
6	*RPS6*	0.464	*TUBb*	0.296	*RPS6*	0.828	*RPL28*	1.110
7	*TUBb*	0.536	*RPL28*	0.314	*GAPDH*	0.828	*TUBb*	1.175
8	*GAPDH*	0.590	*SDHA*	0.474	*TUBb*	0.876	*GAPDH*	1.231
9	*SDHA*	0.657	*GAPDH*	0.510	*UBC*	0.913	*SDHA*	1.294
10	*UBC*	0.723	*SYN6*	0.636	*SDHA*	1.012	*UBC*	1.401
11	*SYN6*	0.781	*UBC*	0.671	*SYN6*	1.181	*SYN6*	1.412
12	*ACTb*	1.027	*ACTb*	1.507	*ACTb*	1.886	*ACTb*	2.443
13	*TUBa*	1.498	*TUBa*	2.768	*TUBa*	3.786	*TUBa*	4.076

### Effect of reference gene selection

**Fig 4:**
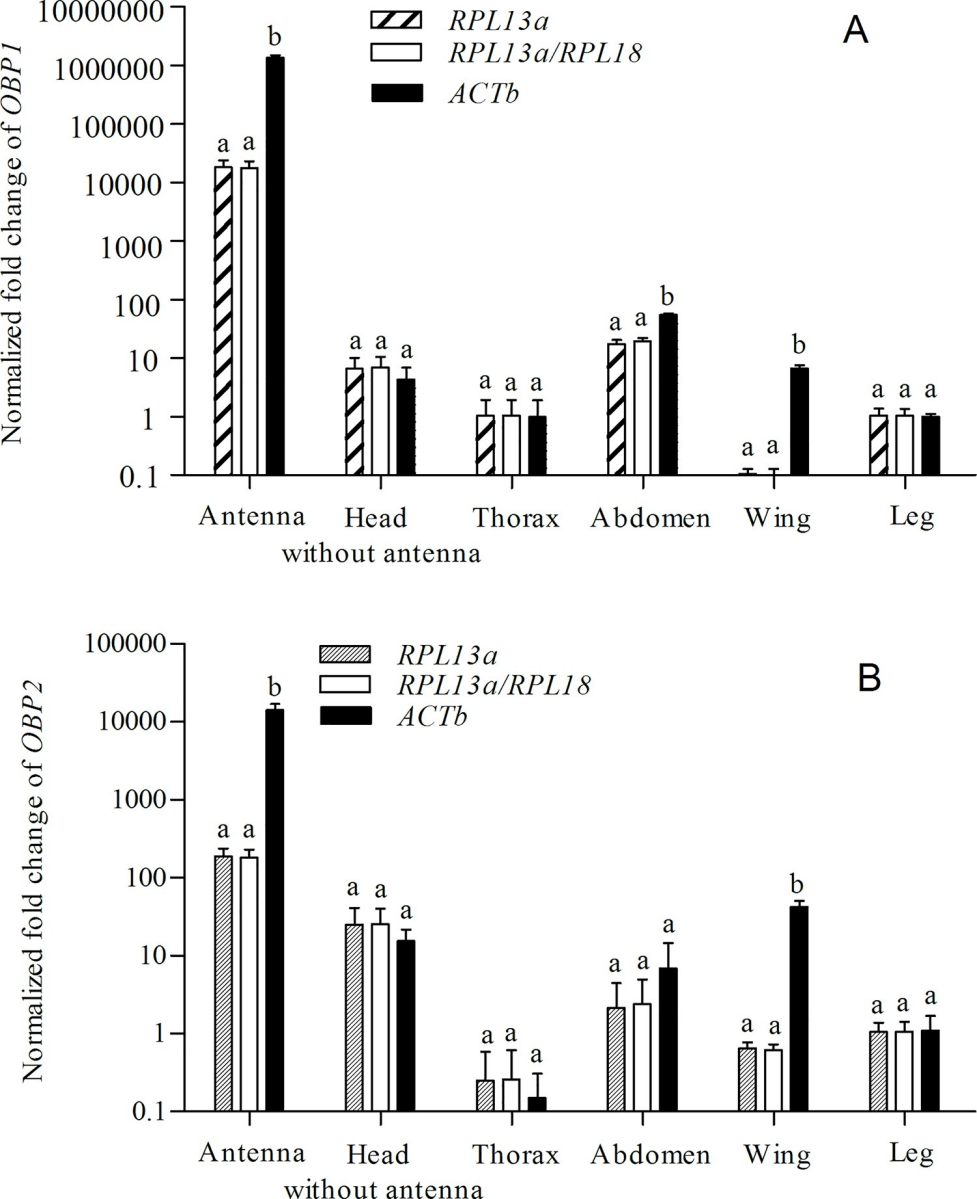
Relative expressions of two odorant-binding protein genes in different tissues normalized by different reference genes. (A) *OBP1*; (B) *OBP2*. Values are means ± SD based on three biological replicates. The expression levels of the legs were used as a calibrator to calculate the relative fold change in the different tissues. Different letters mean significant differences (*P* < 0.05).

To evaluate the reliability of the chosen reference genes in *H*. *oblita*, we selected two odorant-binding protein genes (*OBP1* and *OBP2*) as the target genes across different tissues (Fig 4). Despite its frequent use as a reference gene, *ACTb* ranked next to last among the 13 candidate genes in the different tissues (Fig 3B). The expression profiles of the two target genes normalized with *RPL13a* were consistent with the results of *RPL13a*/*RPL18* (Fig 4). When calculated with *ACTb*, however, the expression levels of *OBP1* in the antennae, abdomen, and wings and *OBP2* in the antennae and wings were overestimated (Fig 4). The unstable reference genes led to more than 70-fold (head without antenna) and 60-fold (wing) errors in the quantification of *OBP1* and *OBP2* (Fig 4). Compared with the normalization results of *RPL13a* and *RPL13a*/*RPL18*, *OBP1* expression in the abdomen normalized with *ACTb* increased by 2.83-fold and 3.14-fold, respectively.

## Discussion

The reliability of qRT-PCR results is determined by components, including sample quality, primer specificity and suitable reference genes [11]. In this study, we obtained all of the samples from fresh insects that were well preserved after processing. We checked all RNA samples by A260/A280, and eliminated the DNA residues by the gDNA eraser in the reagent kit. The primers of the selected genes had good efficiencies, ranging from 91.33% to 103.03% (R^2^>0.99), and the corresponding melting curve contained a single sharp peak (Table 1). Our data suggested that RNA quality and primer design were adequate for qRT-PCR. The expression level of the reference gene should be within a reliable range [34]. The median Ct values were in the range of 17.23 and 25.22, which indicated that they conformed to the basic requirements for qualified reference genes (Fig 1).

The evaluation of reference genes is an important procedure for data normalization [35–37]. *Tribolium castaneum* was the first coleopteran insect in which reference genes were validated [2, 26]. Studies on the selection of reference genes have now been reported for several Coleoptera, including *Agrilus planipennis* [38], *Mylabris cichorii* [39], *Harmonia axyridis* [40], and *Anomala corpulenta* [18]. However, the best reference genes may vary significantly among different insect species. Also, the reference gene can be affected by biotic or abiotic factors, and no reference gene is likely to be suitable for all experiment treatments [41, 42]. *ACTb* is an important component of the cytoskeleton and widely distributed in cells. It has been the most common reference gene used for expression analysis [9, 43, 44]. *ACTb* was selected as a standardized reference gene in *H*. *oblita* [17, 19–21]. Our results showed that *RPL18* was the best reference gene across different tissues of *H*. *oblita* adults, but among the tested genes, *ACTb* was next to last in stability (Fig 3B). We used *OBP1* and *OBP2* to validate the effectiveness of the candidate reference genes. Because of the instability of *ACTb*, the normalized results of the target genes in antennae, abdomen, or wings were overestimated (Fig 4). *ACTb* expression was significantly lower in antennae and wings than in other tissues. Therefore, up to 70-fold (head without antenna) and 60-fold (wing) changes were calculated in the relative expression of *OBP1* and *OBP2*. The results show that the inappropriate selection of reference genes could lead to substantial errors in the quantitation of the target genes. Several other studies have challenged the applicability of *ACTb* as a reference gene for qRT-PCR [45–47]. These earlier results, and those of the present study, indicate that this “classic” gene is variable and needs to be assessed before further use as a reference gene.

Previous studies demonstrated that ribosomal protein genes exhibit high expression stability and should be considered as a source of candidate reference genes. In *Cimex lectularius*, *RPL18* was the best reference gene among different developmental stages and tissues [48]. The expression of *RPL32* was the best reference gene among developmental stages of *Bactrocera* (*Tetradacus*) *minax* [49]. *RPL28* and *RPS15* were the best reference genes under temperature stress [50]. After fungal infection, *RPS3*, *RPS18*, and *RPL13a* expressed stably in *T*. *castaneum* [2]. *RPL10* expression was stable within populations in *Spodoptera litura* [23]. Ribosomal proteins are the main components of the ribosome, which is involved in cell metabolism and regulation [51]. Our results showed that *RPL18 and RPL13a* were always in the top three of the stability ranking, and they were the best pair at different temperatures, developmental stages, and tissues (Fig 3). In some studies, however, ribosomal protein genes had unstable expression [7, 52]. Our study showed that the gene with the most variable expression in all of the experimental conditions was *TUBa* (Fig 3). The M value of *TUBa*, determined by geNorm, was above the cutoff value of 1.5 under all experimental conditions. This finding indicates that it is unsuitable as a reference gene for *H*. *oblita* (Fig 2B). *TUBa* was identified as an available reference gene, however, in *Tetranychus cinnabarinus* [53], *Drosophila melanogaster* [9], and *Nilaparvata lugens* [54].

We used five statistical programs to analyze the stability rankings. The ΔCt method analyzes stability by calculating the pairwise variation within the candidate genes [30]. Ct raw data was used by BestKeeper to determine the stability index by ranking the standard deviation (SD) and coefficient of variance (CV) of each candidate gene [27, 28]. In contrast to BestKeeper, the Ct value should be transformed to linear relative expression for NormFinder and geNorm [31, 32, 55]. For each candidate gene, both programs measure the expression variation in pairs to provide a stability value. Because of the unique analytical methods, the statistical results of the best-ranked genes showed variation among the different tools [56]. *RPS18* and *RPL18* ranked in the top three most stable, across sexes, according to the three methods except for BestKeeper (Table 2). Among the tested temperatures, *RPL13a* and *RPL18* exhibited the great stability using ΔCt, BestKeeper, and geNorm, but in NormFinder, the most stable genes were *SDHA*, *TUBb*, and *GADPH* (Table 2). Despite these slight differences, the overall trends of the four methods were similar in the stability rankings. GeNorm analyzed whether the pairwise variation (V_n_ /V_n+1_) was above 0.15 and determined the optimal number for reliable normalization. In this study, the V_2/3_ value within all factors was below 0.15, which meant that two reference genes were sufficient as internal standards (Fig 2B). Combined with the comprehensive ranking of RefFinder, *RPL13a* and *RPL18* were considered to be the best normalizing genes across all developmental stages, tissues, and temperature treatments; *RPL13a* and *RPS3* across pesticide and photoperiod treatments; and *RPS18* and *RPL18* across sexes (Fig 3).

## Conclusions

We investigated 13 candidate reference genes of *H*. *oblita* under abiotic and biotic stresses and used five algorithms to evaluate their expression stability. We found reasonable variation in stability ranking as a result of the different formulas. RefFinder synthesized the results of these algorithms and provided the final results. Combined with the optimal reference gene number provided by geNorm, *RPL13a* and *RPL18* were the most suitable reference genes in developmental stages, tissues, and temperature treatments; *RPL13a* and *RPS3* in pesticide and photoperiod exposure; and *RPS18* and *RPL18* between the two sexes. *OBP1* and *OBP2* were used as target genes to validate the difference between the selected optimum reference genes and a classic reference gene. The results identified suitable reference genes in *H*. *oblita* under various experimental conditions. These data will benefit future molecular studies.

## Supporting information

S1 Fig Melting curve of 13 candidate reference genes and two target genes. (TIF)
