# Supplementary figures and images for: Evaluation of reference genes for quantitative real-time PCR normalization in the scarab beetle Holotrichia oblita
Source: PLoS One. 2020 Oct 21;15(10):e0240972. doi: 10.1371/journal.pone.0240972 (PMC7577503; doi:10.1371/journal.pone.0240972)

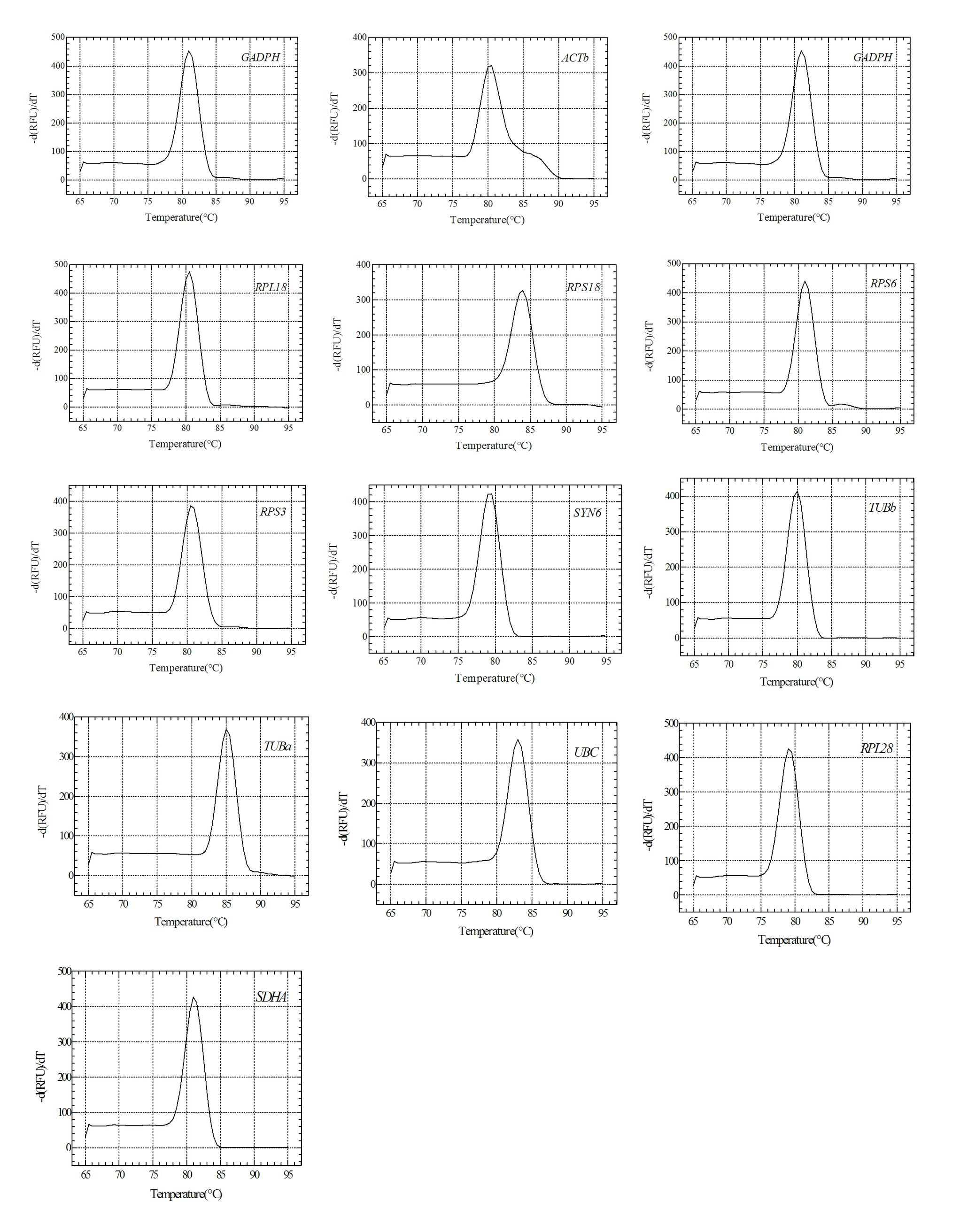

Supplement: S1 Fig — (TIF) [file pone.0240972.s001.tif]
